# Participant Opinions and Expectations about Medical Services at Ultramarathons: Findings from the Ultrarunners Longitudinal TRAcking (ULTRA) Study

**DOI:** 10.7759/cureus.5800

**Published:** 2019-09-29

**Authors:** Martin D Hoffman

**Affiliations:** 1 Physical Medicine and Rehabilitation, University of California, Davis, USA

**Keywords:** blister, emergency medical services, global positioning systems, medical care team, running, wilderness medicine

## Abstract

Background

This work explores the opinions and expectations of ultramarathon runners about medical services and their perceived quality during ultramarathons.

Methods

Focused questions related to medical services at ultramarathons were included in the 2018 survey of Ultrarunners Longitudinal TRAcking (ULTRA) Study enrollees.

Results

Among the 1,156 respondents, 83.2% agreed that ultramarathons should provide at least a minimum level of medical support with basic first aid and emergency transport services rated as the most important medical services, and individuals with basic first aid training rated as the most important medical providers at ultramarathons. Participant safety was felt to largely be the responsibility of each runner as well as the race and/or medical director. Among 832 respondents having completed an ultramarathon in 2016-2018, their impression of medical services at 4,853 ultramarathons was generally favorable. Of the four percent of times in which medical support was needed, it met expectations 74% of the time. Of the total of 240 different medical issues for which medical support was needed, blister management was the most common, accounting for 26.7% of issues.

Conclusions

Even though medical services receive minimal utilization during ultramarathons, ultramarathon runners largely believe that these events should provide at least a minimum level of medical support. Ultramarathon runners place a high onus for safety during ultramarathons on themselves, but they also place a high level of responsibility on race and medical directors, so it is prudent for the race and medical directors to consider this information and avoid a mismatch between runner expectations and the medical services actually provided.

## Introduction

Worldwide participation in ultramarathons (foot races longer than the 42.195 km marathon distance) continues to increase, with around 330,000 individuals accounting for over 530,000 ultramarathon finishes in 2018. Historically attracting largely middle-aged individuals, recent years have seen a relative increase in participation among those between 23 and 35 years of age and an absolute increase in participants over 60 years of age [1]. Younger runners are likely to have less running experience, which has been identified as a risk factor for medical complications during ultramarathons, and older participants are likely to have a higher proportion of the underlying chronic medical conditions that have been documented amongst ultramarathon runners [2, 3-5].

Past work has demonstrated that ultramarathons have tended to have relatively few serious medical issues compared with other sports, though serious and potentially serious medical issues do present [5-14]. Furthermore, the changing demographics of ultramarathon participants may influence the incidence and distribution of medical complications. Thus, some level of medical support at ultramarathons can be justified.

Guidelines about medical support, management of common and potentially life-threatening medical issues, and pre-event medical screening at ultramarathons have been offered previously [15-17]. Presently unknown are the opinions and expectations of ultramarathon runners about medical services, or their perceived quality of experience when seeking medical attention during ultramarathons. This work attempts to explore these issues amongst a large group of ultramarathon runners. It was hypothesized that this group will indicate that safety during an ultramarathon is largely the responsibility of each runner while also believing that at least a minimum level of medical support should be provided at these races. The intention is that consideration of the findings of this work could help race and medical directors avoid a mismatch between participant expectations and the medical services that are provided.

## Materials and methods

Study participants and design

Study participants were enrollees of the Ultrarunners Longitudinal TRAcking (ULTRA) Study. Enrollment for that study has remained open since recruitment was initiated in 2011 via direct electronic mailing to over 3,000 ultramarathon runners, postings on various ultramarathon-related web sites and blogs, advertisements in magazines related to ultramarathon running, and distribution of ﬂyers at a number of the largest ultramarathons in the US. Completion of at least one ultramarathon of 50 km or longer was required for enrollment. Information collected at enrollment via online questionnaire included self-report on a wide range of personal characteristics and exercise history.

The Institutional Review Board of the Veterans Affairs Northern California Health Care System has provided ongoing approval for the ULTRA Study (10-11-00604). Further study details and findings from the enrollment [3, 18-19] and initial follow-up [20] ULTRA Study surveys can be found elsewhere.

Data collection

The data examined herewith are largely from the second follow-up questionnaire that was completed during 2018 by ULTRA Study enrollees. The survey inquired about recent ultramarathon participation and included focused questions related to medical services at ultramarathons starting with the question “Should ultramarathons provide at least some minimum level of medical support?” with answer options of "yes" and "no". Those answering "yes" were asked to rate the importance of various medical services and types of medical providers to be available at ultramarathons on a scale (with numeric rating) of very important (1), important (2), moderately important (3), slightly important (4), and not important (5). Those answering "no" were asked to account for their answer by selecting one or more of five specific options including “it is not necessary because medical issues in ultramarathons are rare”, “it is not necessary because medical issues in ultramarathons are not usually serious”, “it will add to the cost of participating”, “participants should be able to take care of themselves”, and “other” with the option for text entry.

The survey also assessed opinions about whether or not ultramarathons should disclose information about the available medical services and providers, and beliefs about ultramarathons making specified (non-steroidal anti-inflammatory drugs (NSAIDs), acetaminophen and anti-nausea medication) or other medications available during the race. Opinions were assessed about race participants sharing basic medical information (such as history of serious medical issues, medication list, and allergies) with race medical staff prior to ultramarathons, mandating global position system (GPS) tracking devices for ultramarathons in remote environments extending over multiple days, cancellation of ultramarathons for adverse environmental conditions, and interest in pre-race medical and science presentations at ultramarathons in which they participate. Additionally, a series of questions were asked about the medical services that were available, and their use of and satisfaction with those services at each of the ultramarathons that respondents had started in the prior two years.

Statistical analysis

Various characteristics were compared between those with different answers on whether or not at least a minimum level of medical support should be provided at ultramarathons. Group comparisons of nominal data were made with the Mann Whitney test since these data were generally skewed according to the D'Agostino-Pearson normality test. Group comparisons of categorical data were made with the Fisher’s exact test. Importance ratings on aspects of safety and medical services were compared with the Kruskal-Wallis test and Dunns post-test since these data were also skewed. Statistical significance was set at p<0.05.

## Results

Study participants

There were 1,156 study participants responding to the initial questions and 1,135 completed the full survey. Selected characteristics of the group are shown in Table 1. Respondents were largely well-educated, middle-aged men from the US, who were still active at ultramarathon running and had nearly a decade or more of experience at ultramarathon running.

**Table 1:** Selected characteristics of the respondents answering the question “Should ultramarathons provide at least some minimum level of medical support?” Full group n=1,156; respondents who replied "yes" n=962 (83.2%); respondents who replied "no" n=194 (16.8%). Data are presented as median value (with interquartile range) or as a percentage. The p-values are for comparison of the two subgroups. ^a^ Twelve years equates to completion of high school and scale was capped at 18 years (six years or more following completion of high school). ^b^ The question was phrased “Would you like to see more pre-race medical and science presentations at ultramarathons you participate in? Yes or No.”

Characteristic	Full group	Yes-group	No-group	p-value
Age (years)	49 (41-57)	49 (40-57)	49 (42-57)	0.15
Sex (% men)	66.5	64.7	75.8	0.0026
Home country (% from US)	86.8	86.7	87.1	1.00
Schooling completed (years)^a^	18 (16-18)	18 (16-18)	18 (16-18)	0.59
Time since first ultramarathon (years)	10 (8-15)	10 (8-15)	11 (8-16)	0.014
Completed an ultramarathon in 2014-2018 (%)	85.1	84.7	87.1	0.44
Ultramarathons started in prior two years (n)	3 (0-6)	2 (0-6)	4 (0-8)	0.0015
Wanting more pre-race medical/science presentations (%)^b^	65.8	67.6	57.1	0.0058

Opinions about medical services

Most (83.2%) of the respondents answered "yes" to the question “Should ultramarathons provide at least some minimum level of medical support?". Group differences were evident between those with differing answers (Table 1). Specifically, those who were not in favor of medical support compared with the others were more likely (p=0.0026) to be men, to have a statistically longer (p=0.014) ultramarathon running history, to have started roughly twice as many (p=0.0015) ultramarathons in the prior two years, and to have indicated less (p=0.0058) interest in more medical and scientific presentations prior to races. Of those not in favor of medical support, 68.0% reasoned that participants should be able to take care of themselves (Table 2). Among those selecting “other” as an explanation, the largest grouping of text entries (16%) were along the lines that participants can decide to assume the risk as long as it is disclosed that medical services are not provided.

**Table 2:** Explanations provided by those who responded "no" to the question “Should ultramarathons provide at least some minimum level of medical support?” Explanations are provided by 16.8% of respondents. Note that respondents could select multiple reasons.

Explanation	Affirmed (%)
Participants should be able to take care of themselves	68.0
Will add to the cost of participating	23.2
Not necessary because medical issues are not usually serious	11.3
Not necessary because medical issues are rare	7.2
Other	39.7

A very high percentage (91.5%) of the full group of respondents indicated that ultramarathons should make it clear what medical services are available. Among this group, 89.2% indicated that the type of medical providers that are available should also be disclosed. A high percentage (86.4%) of the full group of respondents also agreed that it is appropriate to be asked to share basic medical information with the race medical staff prior to an ultramarathon. Those agreeing were more likely to have indicated that some level of medical support should be provided at ultramarathons (84.3% vs. 76%, p=0.014). Of those indicating it was not appropriate, the main reason, endorsed by 62.3%, was a concern that their privacy may not be protected.

Importance ratings for three aspects of safety and medical services during an ultramarathon are shown in Table 3. Participant safety was felt to largely be the responsibility of each runner as well as the race and/or medical director. The availability of basic first aid and emergency transport were thought to be the most important medical services, and individuals with basic first aid training were felt to be the most important medical providers at ultramarathons.

**Table 3:** Importance ratings by ultramarathon runners for three aspects of safety and medical services during an ultramarathon Ratings were on a scale (with numeric rating) of very important (1), important (2), moderately important (3), slightly important (4), and not important (5). The initial set of responses were by all respondents, whereas the ratings on importance of specific medical services and medical providers were only by the 83.2% of respondents indicating they believed that ultramarathons should provide at least some minimum level of medical support. All pairwise comparisons within a topic differed (p<0.001) except between pairs with the same superscript letters. NSAIDs - nonsteroidal anti-inflammatory drugs

Safety or medical aspect	Importance rating (mean ± SD)
Responsibility for assuring participant safety	
Each participant	1.4 ± 0.7
Race and/or medical director	1.5 ± 0.7
Organizing body for the sport	2.7 ± 1.3^a^
Local authorities	2.7 ± 1.2^a^
Importance of specified medical services	
Basic first aid	1.2 ± 0.5
Emergency transport	1.9 ± 1.0
Wound management	2.4 ± 1.0^a^
Automated external defibrillator (AED)	2.4 ± 1.3^a^
Blister care	2.7 ± 1.3
Intravenous (IV) hydration	3.1 ± 1.3
On-site blood testing for hyponatremia	3.5 ± 1.3^b^
Medications (e.g., NSAIDs, acetaminophen, anti-nausea)	3.5 ± 1.2^b^
On-site blood or urine testing for potential kidney injury	3.6 ± 1.2^b^
Importance of specified medical providers	
Individual with basic first aid training	1.6 ± 0.9
Emergency medical technician (EMT)	2.2 ± 1.2^a^
Paramedic	2.3 ± 1.2^a^
Nurse	2.9 ± 1.2^b^
Person skilled at blister management	3.1 ± 1.3^b^
Physician	3.1 ± 1.3^b^
Massage therapist	4.0 ± 1.1^c^
Physical therapist	4.2 ± 1.0^c^
Podiatrist	4.4 ± 0.9^d^
Chiropractor	4.5 ± 0.8^d^

Over half (59.0%) of the full group of respondents indicated that no medications need to be made available at ultramarathons. Those responding this way were less likely to have indicated that some level of medical support should be provided at ultramarathons (79.6% vs. 88.3%, p<0.0001). Of those not responding this way, most (58.5-66.3%) indicated that acetaminophen, NSAIDs, and anti-nausea medication should be made available (Table 4). The largest grouping of text entries for the 8.1% “other” selections were for epinephrine or antihistamines (2.8%), antidiarrheals (1.3%), and bronchodilators (1.1%).

**Table 4:** Responses about which medications should be made available at ultramarathons Percentages are among the 49% that did not indicate that "no medication should be made available". Note that respondents could select multiple medication choices. NSAIDs - nonsteroidal anti-inflammatory drugs

Medication	Affirmed (%)
Acetaminophen/paracetamol	66.3
NSAIDs	63.8
Anti-nausea medication	58.3
Other	8.1

The full group of respondents was asked “Should a GPS tracking device be mandated for runners to carry at ultramarathons that are in remote environments and extend over multiple days?” The percentage of responses to the answer options of "yes", "no" and "unsure" were 49.6%, 25.1% and 25.3%, respectively. Those answering "yes" were more likely to have indicated that some level of medical support should be provided at ultramarathons (89.2% vs. 73.0%, p<0.0001). Those answering "no" supported that answer largely by endorsing that runners should be able to take care of themselves (69.7%), that runners should be able to avoid getting lost (47.9%), and that GPS tracking would add to the cost of participating in ultramarathon events (30.6%).

The full group of respondents was also asked “Should race and/or medical directors cancel or stop an ultramarathon if environmental conditions might significantly increase the health risks of participants?” and 78.2% answered "yes". Opinions about which environmental conditions would be appropriate to consider cancelling or stopping a race are shown in Table 5.

**Table 5:** Responses to the question “Which environmental conditions would be appropriate to consider cancelling or stopping a race?” Percentages are among the subset of 78.2% answering "yes" to the question “Should race and/or medical directors cancel or stop an ultramarathon if environmental conditions might significantly increase the health risks of participants?” Multiple responses were allowed.

Response options	Affirmed (%)
Nearby wildfire	88.1
Severe smoke	84.0
Flooding	82.2
Blizzard conditions	75.9
Lightning	59.5
Public health crisis taxing the local medical system	49.9
Severe smog	48.2
Extreme heat	43.8
Extreme cold	38.5
Other	6.8

Experience of medical services

There were 832 respondents who provided information on their experiences of medical services at 4,853 ultramarathons in which they had started during the two years before taking the survey (Table 6). Formal medical support was thought to have been available 49.4% of the time. Of the 96% of cases in which medical support was not needed, respondents indicated that the available medical support seemed adequate 60.9% of the time. Of the 4% of times in which medical support was needed, it met expectations 74% of the time. Of the total of 240 different medical issues for which medical support was needed, blister management was the most common, accounting for 26.7% of the issues.

**Table 6:** Observations on medical support from ultramarathon runners who provided information on ultramarathons in which they participated during 2016-2018 Observations are collected from 832 ultramarathon runners who participated in 4,853 ultramarathons. Most (86.5%) of the ultramarathons were in the US and relate to race distances of (or approximating) 50 km (44.2%), 80 km (19.8%), 100 km (10.5%), 161 km (17.8%) and other distances (7.7%). Runs in which medical support was not needed (96%) were directed to question 2, and the 4% of runs in which medical support was needed (accounting for a total of 240 different medical issues) were directed to questions 3 and 4.

Question	Answer (%)
1. Was formal medical support provided?	
Yes	49.4
No	19.9
Unsure	30.7
2. Did the available medical support seem adequate?	
Yes	60.9
No	3.2
Unsure	36.0
3. Did the medical support meet your expectations?	
Yes	74.0
No	13.0
Partially	13.0
4. For what did you need medical support?	
Blister management	26.7
Musculoskeletal injury (sprain, strain, cramping, bruise)	16.3
Wound management	12.9
Nausea	12.5
Dehydration	10.8
Hypothermia	3.8
Hyponatremia	2.5
Asthma	1.7
Other	12.9

## Discussion

Opinions about medical services

This work offers some insight into the opinions and expectations of ultramarathon runners about medical support at ultramarathons. A key finding is that this sizable group of ultramarathon runners largely believes that ultramarathons should provide at least a minimum level of medical support, with basic first aid and emergency transport being of greatest importance.

The small percentage (16.8%) of ultramarathon runners who indicated that ultramarathons should not provide at least a minimum level of medical support, compared with those with the differing opinion, were more likely to be men, to have a longer ultramarathon running history, and to have been more active at ultramarathon running in the prior two years. They were also less interested in more medical and scientific presentations prior to races, although 57.1% still indicated an interest in this type of educational opportunity. These runners largely supported their belief with an indication that race participants should be able to take care of themselves. It was a small percentage that supported their reasoning that medical support should not be provided with an indication that medical issues are rare or usually not serious. Thus, it seems that even this subset of ultramarathon runners does not deny that there are some risks from participating in ultramarathons. They simply believe that each runner assumes the risks of participating and that they should be capable of managing their own needs.

Opinions expressed on whether or not GPS tracking should be mandated at ultramarathons that are in remote environments and extend over multiple days demonstrate the recurring concept among a lot of ultramarathon runners that they should be self-sufficient. While not without some challenges, GPS tracking has been demonstrated to be an effective means of locating a lost runner during a remote ultramarathon [21], so it is unfortunate that only about half of these ultramarathon runners seem to have accepted the potential value of their use as a measure to enhance safety.

While it is evident that this group of ultramarathon runners place a high onus for safety during ultramarathons on themselves, a high level of responsibility is also placed on race and medical directors. It is also evident that accurate disclosure of the available medical services and personnel at ultramarathons is important to participants. This should give pause to race and medical directors to reflect on these expectations since a mismatch between expectations and the medical support actually provided could result in health consequences to participants and represents an important potential liability to events.

A large percentage (78.2%) of the respondents indicated that it would be appropriate to cancel or stop a race if environmental conditions might significantly increase the health risks of participants. It is valuable for the race and medical directors to recognize that they will have a large amount of support for the hard decisions that sometimes need to be made with regard to event cancellation due to environmental issues. Yet, it is evident that this group believes that the environmental conditions warranting race alteration must be quite extreme. It seems bothersome that of those indicating it would be appropriate to alter a race for extreme environmental conditions, less than half (49.9%) affirmed that a public health crisis taxing the local medical system could be an acceptable reason.

Experience of medical services

While medical services were utilized only 4% of the time by this group during the 4,853 ultramarathons in which they had started during the two years before the survey, they generally expressed satisfaction with the level of medical support that was provided. There was also a belief among most (60.9%) of those not needing medical assistance that the medical support was adequate. While over a third (36.0%) were unsure about the adequacy of the medical support, only a very small percentage (3.2%) indicated that medical support was inadequate.

The 4% incidence of seeking medical support amongst this group can be compared with prior studies. For instance, an analysis of the 161-km Western States Endurance Run from 2012 and 2013 events found that 8.2% of starters sought medical consultation, but this did not include blister care [7]. In a much larger analysis of over 26,000 starts at the 56-km Two Oceans Marathon from 2008 through 2011, a 1.3% incidence of medical complications was reported, which was reduced by 39% after a pre-race medical screening and education program was implemented during the next four years [5, 9]. Undoubtedly, numerous factors account for the variations in medical incidents among these studies, inclusive of difference in race distances, course and environmental conditions, level of runner experience and competitiveness, pre-race medical screening, the availability of medical services, and methods for documenting medical encounters [22]. Nevertheless, the present work provides more evidence that the incidence of medical issues in ultramarathons is relatively low.

The most common reason for medical services was blister management, accounting for 26.7% of the medical issues reported in the current analysis. Interestingly, the mean importance ratings for the availability of blister care and for a medical provider trained in blister management were 2.7% and 3.1%, respectively, placing the ratings close to “moderately important”. The fact that the overall utilization of medical care for blister management was quite low (~1%) probably accounts for why the overall importance ratings for such services and personnel was not higher.

Study limitations

Some limitations to the present work are acknowledged. As with any study of this nature, some degree of participation bias is likely. While it is evident that the study participants appeared to be representative of the general population of ultramarathon runners from the past in terms of age, sex and educational distribution, this study had less participation from younger ultramarathon runners than has been evident in some events and when compared with current worldwide participation demographics [1-2, 23-30]. In general, it is also unclear if the study population fully represents the general population of ultramarathon runners in terms of beliefs and expectations about medical services at ultramarathons. The participants were also heavily weighted towards being from the US, which limited the ability to examine geographical differences in opinions about medical services. There is also the potential for reporting bias that could result from the respondents being asked to recall details of events that they participated in as much as two years before the survey.

## Conclusions

From this work, it can be concluded that even though medical services receive minimal utilization during ultramarathons, ultramarathon runners appear to largely believe that these events should provide at least a minimum level of medical support with basic first aid and emergency transport services being perceived as most important. Accurate disclosure of the available medical services and personnel at ultramarathons is also important to this group. Even though they place a high onus for safety during ultramarathons on themselves, a high level of responsibility is also placed on race and medical directors to assure their safety. It is therefore important for the race and medical directors to consider this information and avoid a mismatch between runner expectations and the medical services that are actually provided. Since most of the ultramarathon runners indicated they want more pre-race medical and science presentations, implementation of such programs may be a means to further reduce medical issues in ultramarathons.
